# Supplementary material for: Mechanisms governing the pioneering and redistribution capabilities of the non-classical pioneer PU.1
Source: Nat Commun. 2020 Jan 21;11:402. doi: 10.1038/s41467-019-13960-2 (PMC6972792; doi:10.1038/s41467-019-13960-2)
Supplement: Supplementary file 7 — Source data [file 41467_2019_13960_MOESM7_ESM.zip › Source_Data/FigureS5/FigureS5A_MotifsCommonPeaks/homerResults/motif17.similar.html]

motif17

## Information for motif17

A
C
G
T
A
G
T
C
C
G
A
T
A
C
G
T
C
G
T
A
A
C
G
T
A
G
T
C
C
G
A
T
  
Reverse Opposite:  

G
C
T
A
A
C
T
G
C
G
T
A
A
C
G
T
C
G
T
A
C
G
T
A
T
C
A
G
T
G
C
A
  

|  |  |
| --- | --- |
| p-value: | 1e-95 |
| log p-value: | -2.203e+02 |
| Information Content per bp: | 1.854 |
| Number of Target Sequences with motif | 1321.0 |
| Percentage of Target Sequences with motif | 14.87% |
| Number of Background Sequences with motif | 2834.0 |
| Percentage of Background Sequences with motif | 7.46% |
| Average Position of motif in Targets | 99.2 +/- 54.3bp |
| Average Position of motif in Background | 101.5 +/- 67.4bp |
| Strand Bias (log2 ratio + to - strand density) | 0.0 |
| Multiplicity (# of sites on avg that occur together) | 1.10 |
| Motif File: | file (matrix) reverse opposite |

### Similar de novo motifs found

|  |  |  |  |  |  |  |  |
| --- | --- | --- | --- | --- | --- | --- | --- |
| Rank | Match Score | Redundant Motif | P-value | log P-value | % of Targets | % of Background | Motif file |
| 1 | 0.920 | A G T C A C G T A C G T C G T A A C G T A G T C C G T A A C T G C A G T | 1e-92 | -212.682504 | 15.88% | 8.32% | motif file (matrix) |
| 2 | 0.967 | C G T A A C T G C G T A A C G T C G T A C G T A T C A G | 1e-90 | -207.446732 | 18.11% | 10.10% | motif file (matrix) |
| 3 | 0.925 | A C G T A G T C A C G T C G A T T C G A G A C T A G T C A C G T A T G C C A T G | 1e-60 | -139.413399 | 6.73% | 2.82% | motif file (matrix) |
| 4 | 0.912 | C G A T A C T G G T C A C A G T C T G A C G T A A T C G T G C A C A G T G T C A | 1e-44 | -103.396571 | 3.31% | 1.07% | motif file (matrix) |
